# Supplementary material for: Population genomics and antimicrobial resistance dynamics of Escherichia coli in wastewater and river environments
Source: Commun Biol. 2021 Apr 12;4:457. doi: 10.1038/s42003-021-01949-x (PMC8041779; doi:10.1038/s42003-021-01949-x)
Supplement: Supplementary file 2 — Supplementary Information [file 42003_2021_1949_MOESM2_ESM.pdf]

SUPPLEMENTARY INFORMATION

Population genomics and antimicrobial resistance dynamics of *Escherichia coli* in wastewater and river environments

Running title: Genomic comparison of pan-aminoglycoside resistant *E. coli* from wastewater and rivers

Jose F. Delgado-Blas[1], Cristina M. Ovejero[1], Sophia David[2], Natalia Montero[1], William Calero-Caceres[3,4], María Pilar Garcillan-Barcia[5], Fernando de la Cruz[5], Maite Muniesa[3], David M. Aanensen[2], Bruno Gonzalez-Zorn[1*]

[1] Antimicrobial Resistance Unit (ARU), Animal Health Department, Faculty of Veterinary Medicine and VISAVET, Complutense University of Madrid, Madrid, Spain
[2] Centre for Genomic Pathogen Surveillance (CGPS), Wellcome Sanger Institute, Hinxton, United Kingdom
[3] Department of Genetics, Microbiology and Statistics, Faculty of Biology, University of Barcelona, Barcelona, Spain
[4] UTA RAM One Health, Faculty of Food Science, Engineering and Biotechnology, Technical University of Ambato, Ambato, Ecuador (Current address)
[5] Institute of Biomedicine and Biotechnology (IBBTEC), CSIC, University of Cantabria, Santander, Spain

***Corresponding author:**
Name: Bruno Gonzalez-Zorn
Address: Antimicrobial Resistance Unit, Animal Health Department, Faculty of Veterinary Medicine and VISAVET, Complutense University of Madrid, Av. Puerta de Hierro, Madrid, 28040
Telephone number: +34 913943707
E-mail address: bgzorn@ucm.es

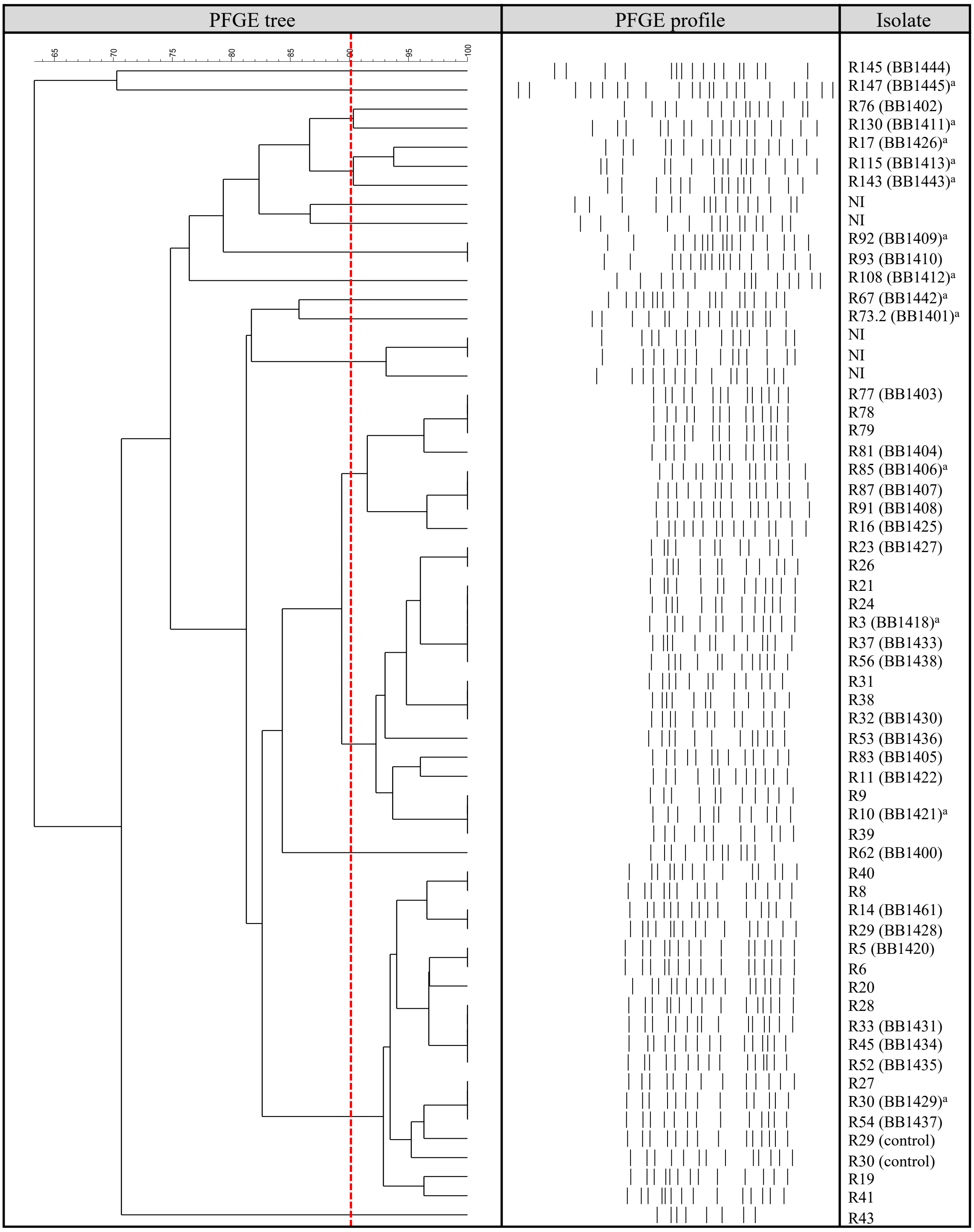

Supplementary Figure 1. Pulsed Field Gel Electrophoresis (PFGE) analysis of all aminoglycoside-resistant *E.* coli. Isolates with ambiguous PFGE profile were not included. Clone is defined by a cutoff of ≥90% similarity. *Escherichia coli* isolates with BB14XX code were selected for WGS by Illumina technology. [a] *E. coli* isolates sequenced by Nanopore technology. NI: isolates not included in this work. Control: duplicate isolate for PFGE internal evaluation.

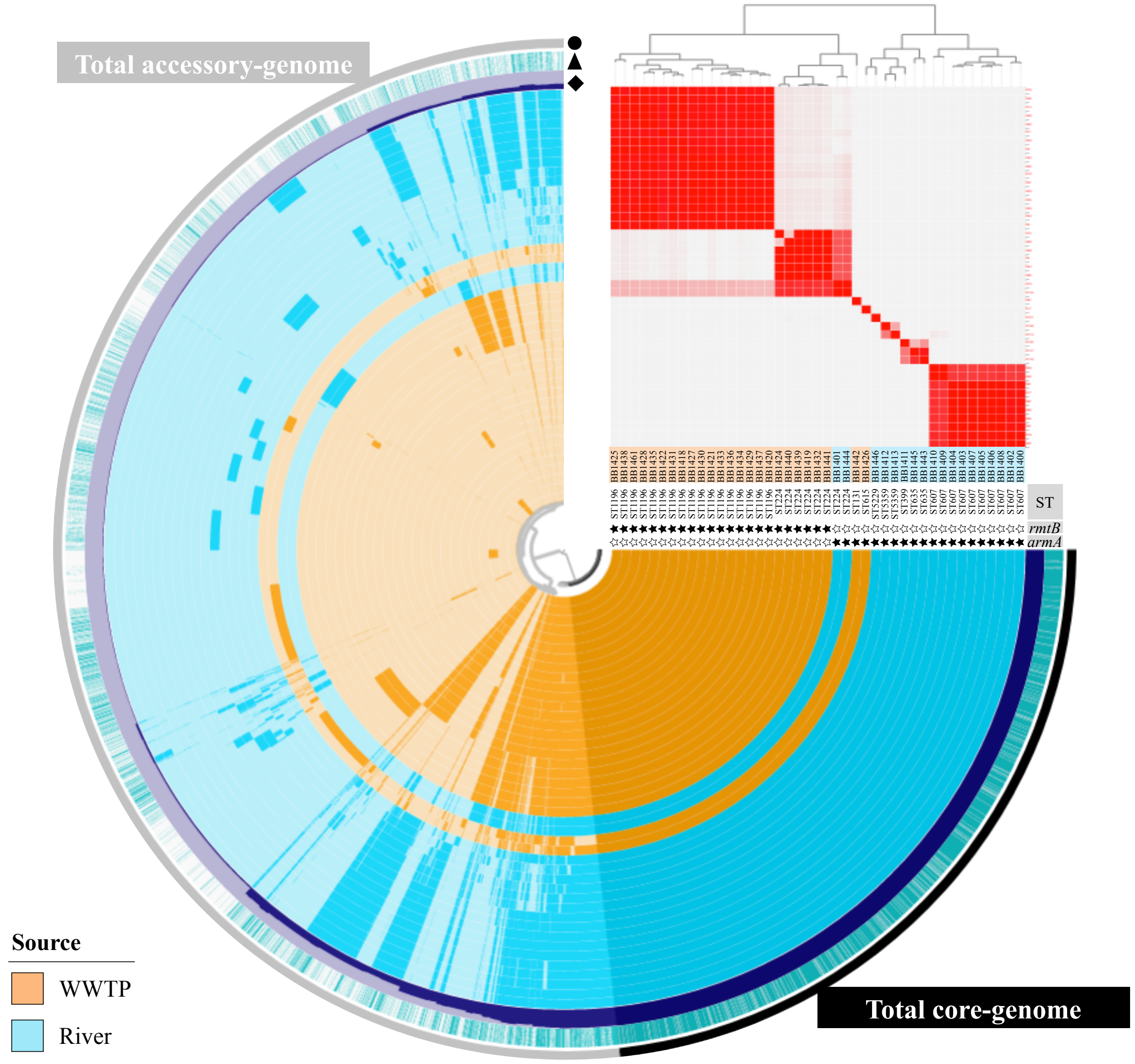

Supplementary Figure 2. Pan-genome *E. coli* analysis. The source of the isolates is indicated by colors in the name of the isolate and the circular diagram. *E. coli* ST is indicated in the tips, as well as the presence (★) and the absence (☆) of 16S-RMTas genes. Pan-genome tree distribution is based on the presence/absence of all predicted gene clusters. Heat map shows the average nucleotide identity across all genomes (range 0.99 to 1.0). Circular diagram shows the presence (dark color) and absence (light color) of gene clusters. Inner ring (◆) indicates the number of genomes that possess the gene cluster. Middle ring (▲) indicates the gene clusters with associated known function. Outer ring (●) indicates the gene clusters belonging to core-genome (black) and to accessory-genome (gray).

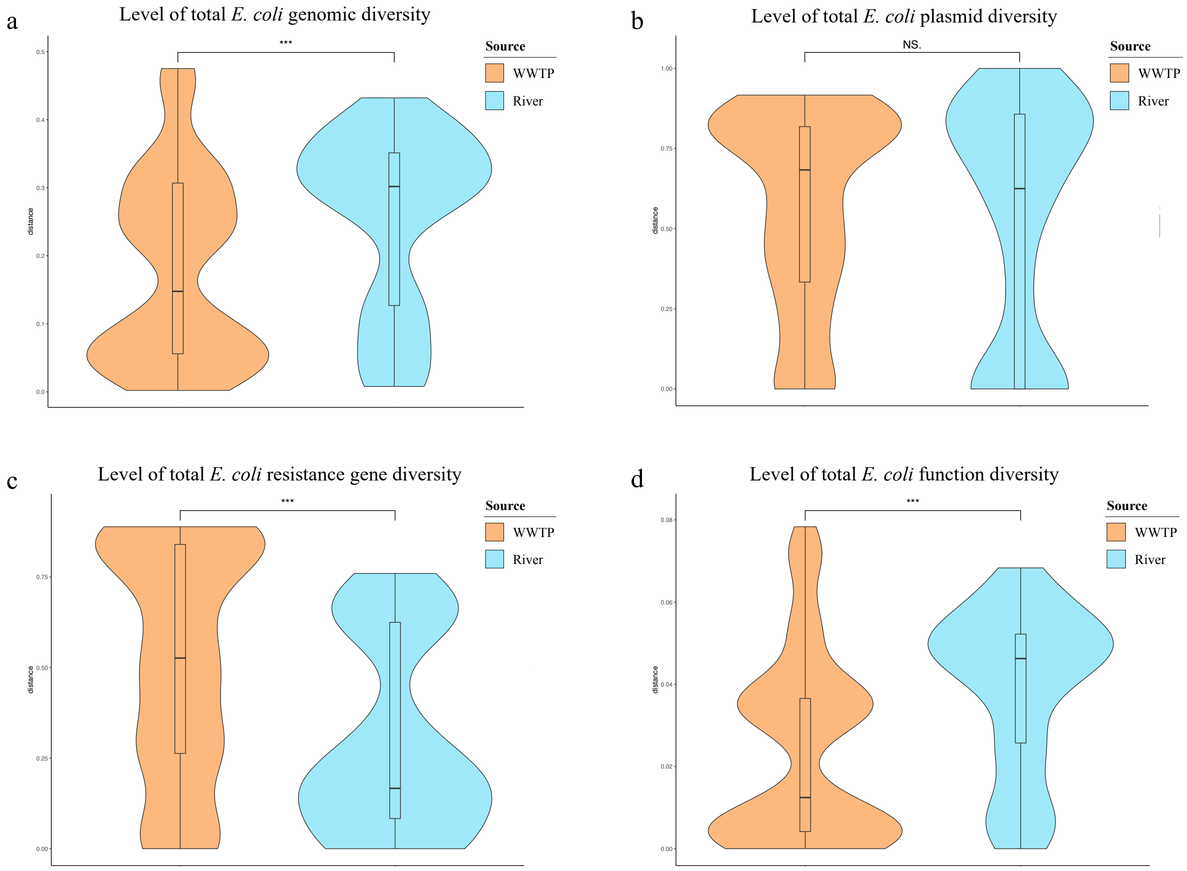

Supplementary Figure 3. Violin plots showing medians and quartiles for diversity levels of *E. coli* populations according to Jaccard distance index and the environment. *E. coli* populations from both WWTP environments (n=25 independent isolates) and river environments (n=18 independent isolates) are indicated by colors. Highly-significant differences are indicated by ***. Non-significant differences are indicated by NS. **a:** Level of total genomic diversity based on core-genome analysis. **b:** Level of total plasmid diversity based on plasmid incompatibility groups. **c:** Level of total antibiotic resistance gene diversity. **d:** Level of total function diversity based on function prediction from core-genome analysis.

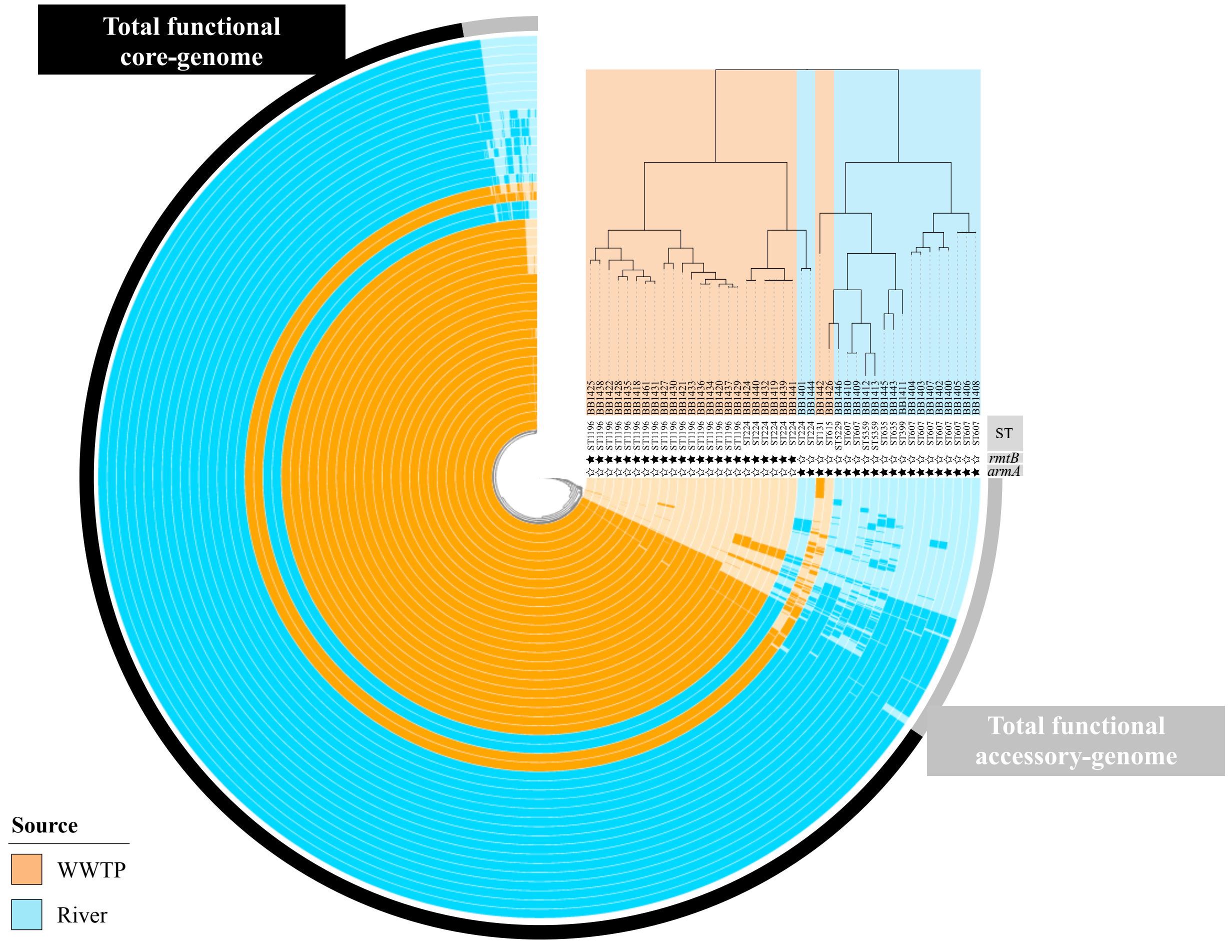

Supplementary Figure 4. Functional pan-genome *E. coli* analysis. The origin of the isolates is indicated by colors in the tree and the circular diagram. *E. coli* ST is indicated in the tips, as well as the presence (★) and the absence (☆) of 16S-RMTas genes. Functional pan-genome tree distribution is based on the presence/absence of all predicted functions. Circular diagram shows the presence (dark color) and absence (light color) of predicted functions. Outer ring indicates the predicted functions belonging to functional core-genome (black) and to functional accessory-genome (gray).

a

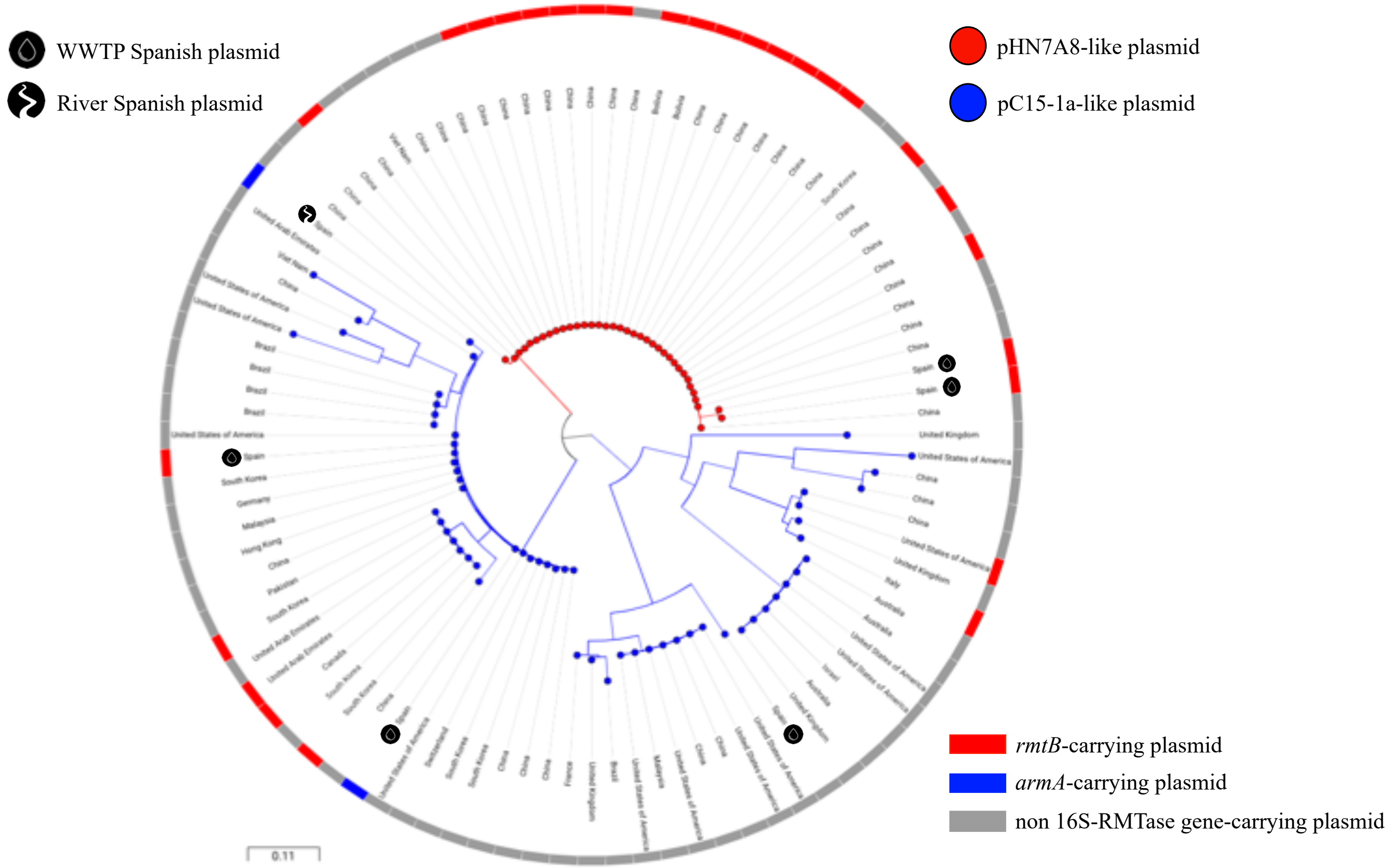
WWTP Spanish plasmid
River Spanish plasmid
pHN7A8-like plasmid
pC15-1a-like plasmid
rmtB-carrying plasmid
armA-carrying plasmid
non 16S-RMTase gene-carrying plasmid

b

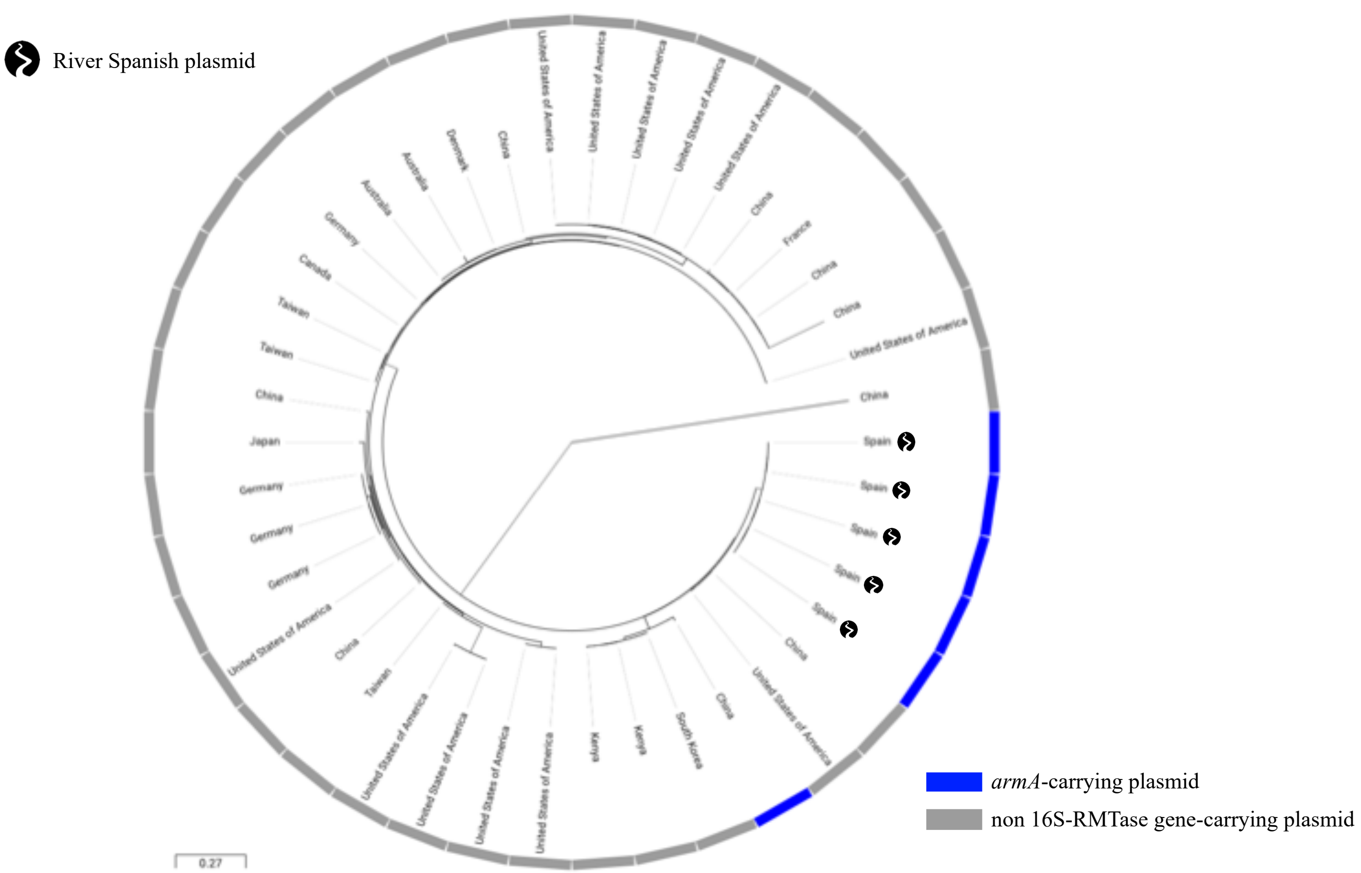

Supplementary Figure 5. Total SNP-trees of most predominant 16S-RMTase gene-carrying plasmids in waters of Barcelona, including all plasmids with identical origin of replication and complete metadata from other sequencing projects around the world. Country of origin is indicated in the tips of the SNP-tree. Spanish plasmids and their sources (WWTP or river) are shown by symbols. The presence/absence of 16S-RMTase gene in the plasmids is indicated by color in the outer ring. **a:** IncFII plasmid type SNP-tree. pHN7A8-like and pC15-1a-like plasmids are differentiated by colored branches and nodes in the SNP-tree. Interactive visualization of IncFII SNP-tree with all associated metadata is available at https://microreact.org/project/yeLN26yJf. **b:** IncHI2A plasmid type SNP-tree. Interactive visualization of IncHI2A SNP-tree with all associated metadata is available at https://microreact.org/project/yNTuhk1Jw.

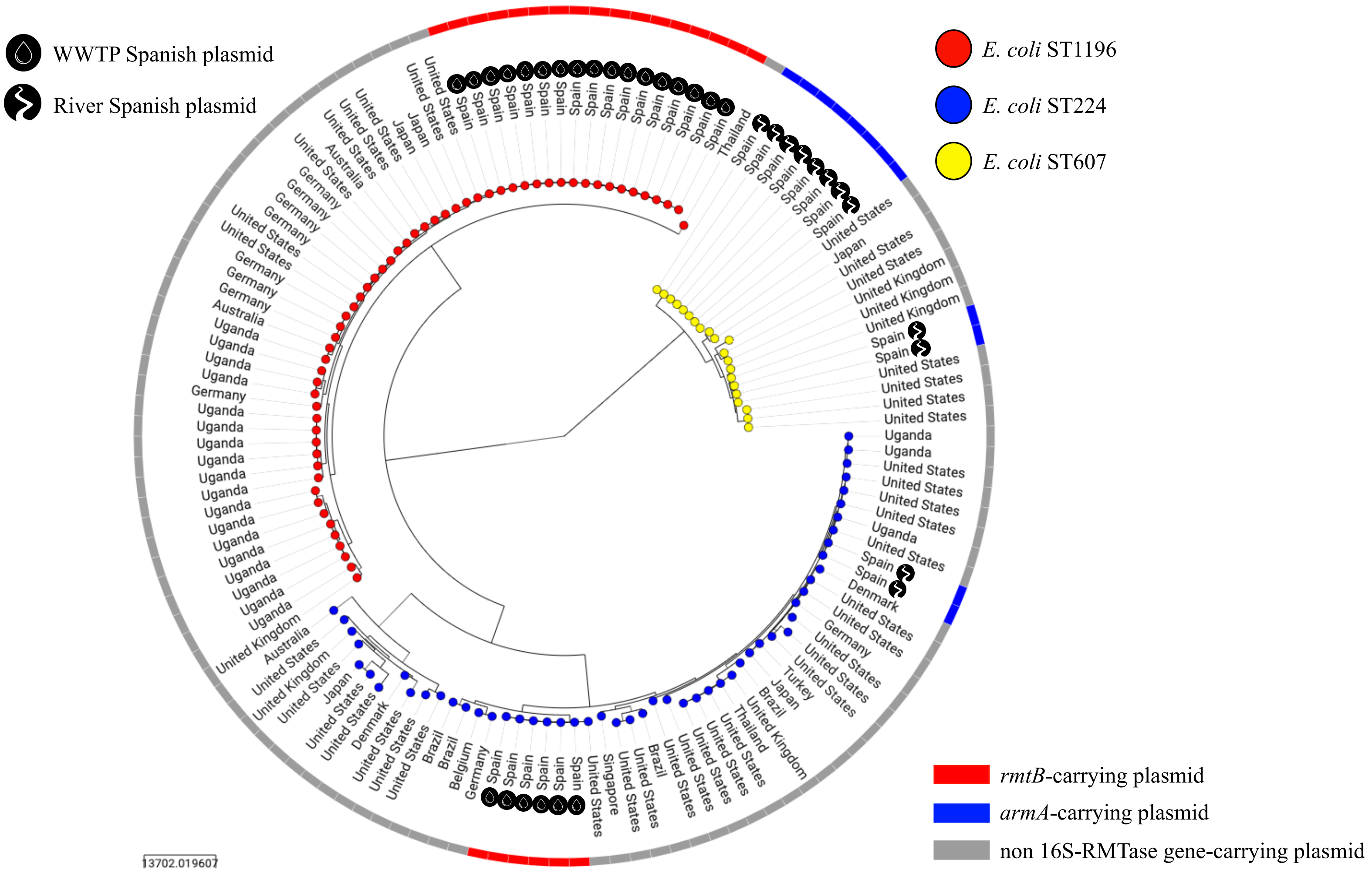

Supplementary Figure 6. Total chromosome SNP-tree of most predominant 16S-RMTase gene-carrying *E. coli* STs in waters of Barcelona, including all *E. coli* with identical sequence type and complete metadata from other sequencing projects around the world. Country of origin is indicated in the tips of the SNP-tree. Spanish *E. coli* isolates and their sources (WWTP or river) are shown by symbols. The presence/absence of 16S-RMTase gene in the isolates is indicated by color in the outer ring. *E. coli* STs are differentiated by colored branches and nodes in the SNP-tree. Interactive visualization of *E. coli* SNP-tree with all associated metadata is available at https://microreact.org/project/a9-1ZIze0.

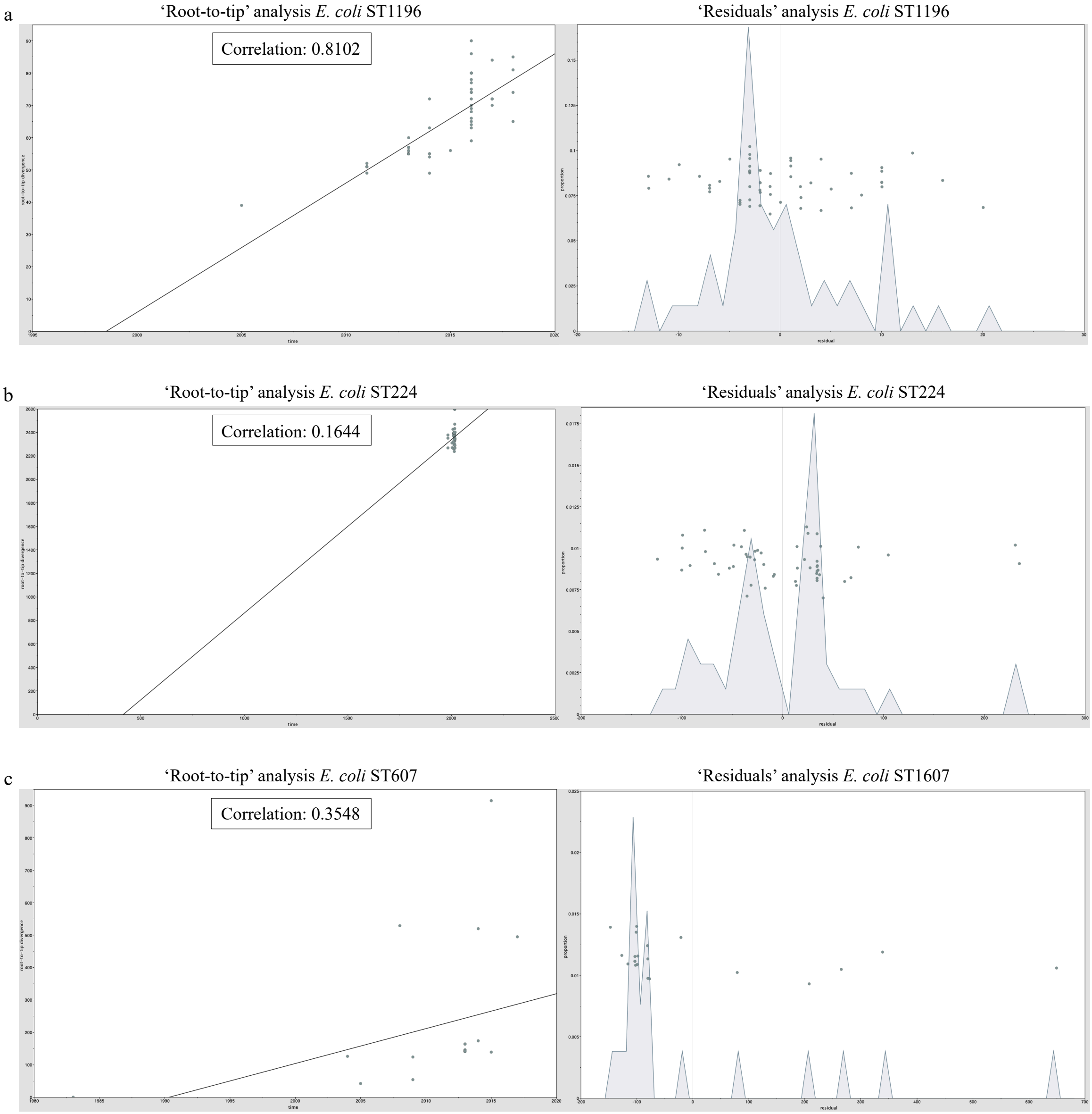

Supplementary Figure 7. 'Root-to-tip' regression analysis and related 'residuals' plots for the three most predominant 16S-RMTase gene-carrying *E. coli* STs in waters of Barcelona, including all *E. coli* with identical sequence type and complete metadata from other sequencing projects around the world. 'Root-to tip' panels show the linear regression for each *E. coli* sequence type adjusted to the 'best-fitting-root', taking into account the sampling date of the isolates and indicating the value for the correlation coefficient. 'Residuals' plots show the histogram and scatterplot of the residuals of the linear regression for each *E. coli* sequence type. **a:** Temporal signal analysis of *E. coli* ST1196. **b:** Temporal signal analysis of *E. coli* ST224. **c:** Temporal signal analysis of *E. coli* ST607.
