# Supplementary material for: Population genomics and antimicrobial resistance dynamics of Escherichia coli in wastewater and river environments
Source: Commun Biol. 2021 Apr 12;4:457. doi: 10.1038/s42003-021-01949-x (PMC8041779; doi:10.1038/s42003-021-01949-x)
Supplement: Supplementary file 3 — Description of Additional Supplementary Files [file 42003_2021_1949_MOESM3_ESM.pdf]

Description of Additional Supplementary Files

File name: Supplementary Movie 1

Description: Movie of *E. coli* ST1196 spatio-temporal dissemination worldwide. The movie was generated from phylogeographic analysis of SNPs from non-recombinogenic regions of 40 ST1196 *E. coli* chromosomes.

File name: Supplementary Data 1

Description: Data of all bacteria highly resistant to aminoglycosides from wastewater treatment plants and rivers of Barcelona (Spain). *Escherichia coli* isolates with BB14XX code were selected for WGS by Illumina technology. [a] *E. coli* isolates sequenced by Nanopore technology. Wastewater treatment plant (WWTP) source is designated according to the name of the WWTP and the number of wastewater tank. River source is classified according to river water (RW) and river sediments (RS) and nominated by the name of the river and the sampling location (A or B). Antibiotics tested by disk diffusion method (concentration in mg per disk): AMP (ampicillin), AMC (amoxicillin-clavulanic acid), CTX (cefotaxime), CXM (cefuroxime), ATM (aztreonam), CIP (ciprofloxacin), TET (tetracycline), FOF (fosfomycin), SXT (trimethoprim-sulfamethoxazole), MEM (meropenem), APR (apramycin). Phenotypic susceptibility is indicated by S and phenotypic resistance is indicated by R. Gene refers to 16S-RMTase genes detected.

File name: Supplementary Data 2

Description: KML file to visualize an interactive spatio-temporal reconstruction of *E. coli* ST1196 dissemination worldwide in Google Earth website. The file was derived from phylogeographic analysis of SNPs from non-recombinogenic regions of 40 ST1196 *E. coli* chromosomes.

File name: Supplementary Data 3

Description: Metadata and data of raw reads from Illumina whole genome sequencing of selected *E. coli* from wastewater treatment plants (WWTPs) and rivers of Barcelona (Spain). These data are encompassed under the umbrella project PRJEB34801 in the European Nucleotide Archive (ENA).

File name: Supplementary Data 4

Description: Metadata and data of assemblies from Illumina whole genome sequencing of selected *E. coli* from wastewater treatment plants (WWTPs) and rivers of Barcelona (Spain). These data are encompassed under the umbrella project PRJEB34801 in the European Nucleotide Archive (ENA).

File name: Supplementary Data 5

Description: Metadata and data of raw reads from Nanopore whole genome sequencing of selected *E. coli* from wastewater treatment plants (WWTPs) and rivers of Barcelona (Spain). These data are encompassed under the umbrella project PRJEB34801 in the European Nucleotide Archive (ENA).

File name: Supplementary Data 6

Description: Metadata and data of assemblies from Illumina and Nanopore whole genome sequencing of selected *E. coli* from wastewater treatment plants (WWTPs) and rivers of Barcelona (Spain). These data are encompassed under the umbrella project PRJEB34801 in the European Nucleotide Archive (ENA).

File name: Supplementary Data 7

Description: Metadata of all *E. coli* assemblies deposited in Enterobase database belonging to the predominant 16S rRNA methyltransferase gene-carrying sequence types found in waters of Barcelona (ST1196, ST224 and ST607). Only the isolates with comprehensive metadata were included in the study. The location of isolates without latitude and longitude information was stablished in a central point of the country of origin. The presence/absence of 16S rRNA methyltransferase genes, as well as the presence/absence of the main plasmid types involved in the dissemination of these genes in waters of Barcelona, is indicated by either detected (D) or non-detected (ND).

File name: Supplementary Data 8

Description: Metadata of all plasmids deposited in NCBI database with an origin of replication identical to the predominant 16S rRNA methyltransferase gene-carrying plasmid types found in waters of Barcelona (pHN7A8-like, pC15-1a-like and R478-like). Only the plasmids with comprehensive metadata were included in the study. The location of plasmids without latitude and longitude information was stablished in a central point of the country of origin. The presence of 16S rRNA methyltransferase genes is indicated by the name of the gene, whereas the absence of all possible genes is indicated by non-detected (ND).
