# Supplementary material for: Population genomics and antimicrobial resistance dynamics of Escherichia coli in wastewater and river environments
Source: Commun Biol. 2021 Apr 12;4:457. doi: 10.1038/s42003-021-01949-x (PMC8041779; doi:10.1038/s42003-021-01949-x)
Supplement: Supplementary file 13 — Reporting Summary [file 42003_2021_1949_MOESM13_ESM.pdf]

nature research

Corresponding author(s): Bruno Gonzalez-Zorn

Last updated by author(s): Feb 16, 2021

Reporting Summary

Nature Research wishes to improve the reproducibility of the work that we publish. This form provides structure for consistency and transparency in reporting. For further information on Nature Research policies, see Authors & Referees and the Editorial Policy Checklist .

Statistics

For all statistical analyses, confirm that the following items are present in the figure legend, table legend, main text, or Methods section.

n/a	Confirmed	
☐	☒	The exact sample size (n) for each experimental group/condition, given as a discrete number and unit of measurement
☐	☒	A statement on whether measurements were taken from distinct samples or whether the same sample was measured repeatedly
☐	☒	The statistical test(s) used AND whether they are one- or two-sided<br>*Only common tests should be described solely by name; describe more complex techniques in the Methods section.*
☐	☒	A description of all covariates tested
☐	☒	A description of any assumptions or corrections, such as tests of normality and adjustment for multiple comparisons
☐	☒	A full description of the statistical parameters including central tendency (e.g. means) or other basic estimates (e.g. regression coefficient) AND variation (e.g. standard deviation) or associated estimates of uncertainty (e.g. confidence intervals)
☐	☒	For null hypothesis testing, the test statistic (e.g. F, t, r) with confidence intervals, effect sizes, degrees of freedom and P value noted<br>*Give P values as exact values whenever suitable.*
☐	☒	For Bayesian analysis, information on the choice of priors and Markov chain Monte Carlo settings
☒	☐	For hierarchical and complex designs, identification of the appropriate level for tests and full reporting of outcomes
☒	☐	Estimates of effect sizes (e.g. Cohen's d, Pearson's r), indicating how they were calculated

Our web collection on statistics for biologists contains articles on many of the points above.

Software and code

Policy information about availability of computer code

Data collection	- EnteroBase database version 1.1.2: E. coli genome search was conducted based on E. coli ST and filtered according to available metadata and sequencing quality.<br>- National Center for Biotechnology Information (NCBI) database (Nov-2018): Plasmid search was conducted based on 100% identical origin of replication and filtered according to available metadata and structure completeness.
Data analysis	- Bionumerics version 6.0<br>- FastQC version 0.11.3<br>- Trimmomatic version 0.33<br>- SPAdes version 3.11.0<br>- QUAST version 4.6.0<br>- Prokka version 1.5<br>- MLSTcheck tool version 1.007<br>- ARIBA version 2.12.1<br>- ResFinder database (Oct-2018)<br>- PlasmidFinder database (Oct-2018)<br>- iTOL version 4.4.2<br>- Roary version 3.11.2<br>- SNP-sites tool version 2.4.1<br>- RAxML version 8.2.8<br>- FigTree version 1.4.3<br>- Phandango version 1.3.0<br>- anvi’o version 5.5<br>- Prodigal version 2.6.3

- NCBI's Cluster of Orthologous Groups (COG) database (Mar-2019)
- PyANI version 0.2.7
- R version 3.6.1
- RStudio version 1.2.1335
- vegan package version 2.5-5
- MinKNOW version 2.2.8
- Epi2Me version 3.8.0
- Porechop version 0.2.3
- Unicycler version 0.4.0
- Bandage version 0.8.1
- Progressive Mauve version 2.3.1
- Geneious version 8.1.9
- 'dnadiff' module version 1.3 for nucmer
- BLAST Ring Image Generator (BRIG) version 0.95
- EasyFig version 2.2.2
- MacSyFinder version 1.0.5
- Sequence Mapping and Alignment Tool (SMALT) version 0.7.4
- Microreact version 8.0.0
- Gubbins version 1.4.10
- TempEst version 1.5.1
- Bayesian Evolutionary Analysis Sampling Trees (BEAST) version 2.5.1
- Tracer version 1.7.1
- DensiTree version 2.2.6
- Spatial Phylogenetic Reconstruction of Evolutionary Dynamics (SPREAD) version 1.0.7
- Google Earth Pro version 7.3.2.5776

For manuscripts utilizing custom algorithms or software that are central to the research but not yet described in published literature, software must be made available to editors/reviewers. We strongly encourage code deposition in a community repository (e.g. GitHub). See the Nature Research guidelines for submitting code & software for further information.

Data

Policy information about availability of data

All manuscripts must include a data availability statement. This statement should provide the following information, where applicable:

- Accession codes, unique identifiers, or web links for publicly available datasets
- A list of figures that have associated raw data
- A description of any restrictions on data availability

All raw and assembled sequence data generated during the current study are available in the European Nucleotide Archive (ENA) at EMBL-EBI under the umbrella project PRJEB34801, and properly specified within the paper and its Supplementary Material files. The authors declare that sequence data from public repositories analyzed during the current study are available within the paper and its Supplementary Material files. All other relevant data are available from the corresponding authors.

Field-specific reporting

Please select the one below that is the best fit for your research. If you are not sure, read the appropriate sections before making your selection.

☐ Life sciences ☐ Behavioural & social sciences ☒ Ecological, evolutionary & environmental sciences

For a reference copy of the document with all sections, see nature.com/documents/nr-reporting-summary-flat.pdf

Ecological, evolutionary & environmental sciences study design

All studies must disclose on these points even when the disclosure is negative.

Study description	This work was a descriptive and comparative epidemiological study focused on the analysis of the bacterial, plasmidic and genetic structure of Escherichia coli populations harboring diverse antimicrobial resistance genes from both wastewater and river water samples collected in the area of Barcelona (Spain).
Research sample	Samples from two aquatic environments were collected: wastewater and river water. Wastewater samples were obtained from two different wasterwater treatment plants (WWTP) located in the region of Barcelona. The E. coli isolates from these samples were considered representative of bacterial populations influenced by human activities. River water samples were collected from the water and sediments of two rivers located in the same region that wastewater samples, in order to avoid bias factors related to sampling location. The E. coli isolates recovered fro these samples were considered representative of bacterial populations influenced by natural conditions. The research samples were selected in order to establish further comparisons between the E. coli populations from these two different ecological niches.
Sampling strategy	A total of 8 samples were obtained from two rivers, the Cardener and the Llobregat. From Llobregat river, samples were taken at two locations: from surface water (3 samples on different dates from location A and 1 sample from location B) and sediments (1 sample from each location). From Cardener river, two samples were collected, one from surface water and one from river sediments. For

analysis, 10 mL of river water and 5 g of river sediments were processed. Samples were taken from the high-middle course of the rivers in order to avoid anthropogenic influence over river samples.
A total of 5 wastewater samples of 10 mL were obtained from two WWTPs located in Baix Llobregat (the area where the Llobregat river flows into the Mediterranean). From El Prat WWTP, 3 samples were obtained, and 2 samples were obtained from Gavà WWTP, from different water tanks and on different dates.
Sample strategy was designed in order to obtain a representative bacterial population from the two studied ecological niches, obtaining samples from two well-characterized rivers and WWTPs, at different locations and on different dates of the same temporal frame. Due to the descriptive nature of the current study, sample sizes were sufficient to carry out subsequent analyses.
The specific collection point for each sample and the resulting bacterial isolates recovered from the samples is stated in the Supplementary Material provided with the manuscript.

Data collection

Data collection was directly compiled by members of the Department of Genetics, Microbiology and Statistics of the Faculty of Biology of the University of Barcelona, who were the reponsible team for sample collection. As participants of the current study, the members of team are listed as coauthors.

Timing and spatial scale

All samples from diferent aquatic environments were collected from July to November 2013 (5 months range). Samples of the same river/WWTP were taken from different locations and with at least one month distant over the timeline. The sampling timing was designed and established to obtain a representative bacterial population from each ecological niche on the same temporal frame, in order to avoid discrepancies between samples caused by seasonal changes. However, the 1-month temporal distance between samples from the same river/WWTP was enough to exclude possible bias due to particular conditions.
The specific collection day for each sample and the resulting bacterial isolates recovered from the samples is stated in the Supplementary Material provided with the manuscript.

Data exclusions

No data were excluded from the analyses.

Reproducibility

As the current work was a descriptive study of environmental bacterial populations collected in a restricted spatio-temporal frame, no replicates nor serial measures were taken into consideration. However, bioinformatic pipelines and statistical models applied based on genomic data from sequenced bacterial isolates were tested multiple times with different parameter values in order to verify the reproducibility of the analyses.
Customed R codes designed for comparing the level of different genetic diversities between sequence types (STs) of the same bacterial species from different water environments were proved with random sampling approach before the adjusment and stablishment of the final statistical models.
All analyses and pipelines are extensively explained in the manuscript and customed R codes are publicly available at GitHub (https://github.com/JoseFranciscoDelgadoBlas/bacterial_population_diversity) and Zenodo (DOI: 10.5281/zenodo.4542907) repositories.

Randomization

Samples and, therefore, bacterial isolates recovered from water samples were allocated into two groups according to the source of the sample: wastewater environment (for samples and bacteria obtained from WWTP samples) and river environment (for samples and bacteria obtained from river samples). As previously explained, covariates were controlled by collecting the samples from both different ecological niches in the same general spatio-temporal frame, which minimised the impact of temporal and geographical bias between samples. However, samples assigned to each group were collected from two WWTPs and two rivers, in different locations and on different dates for each WWTP/river, which minimised the bias of particular conditions for specific sampling points and times and, therefore, the impact over the posterior anlyses.

Blinding

Blinding was not relevant to data acquisition and analysis carried out during the current study. As this work was a descriptive and comparative epidemiological study of antimicrobial-resistant bacterial populations from different aquatic environments, no blinding was required. Data from bacterial isolates were collected considering the classification according to the sample source and analyses were carried out based on this classification for comparative purposes.

Did the study involve field work? ☒ Yes ☐ No

Field work, collection and transport

Field conditions

Mean values for parameters measured during sample collection from river environments included: 7.55 m3/s streamflow, 18.6ºC average temperature, 108.8 mm accumulated precipitation and 8.21 water pH. Samples were collected 24-48 hours after rainfall events.
Values for relevant parameters registered from both sampled WWTPs during the sampling period of the current study are available at: http://aca.gencat.cat/web/.content/20_Aigua/02_infraestructures/05_estacions_depuradores_daigues_residuals/Fitxes_EDAR/dpdl_edar_pratdellobregat.pdf for El Prat WWTP, and http://aca.gencat.cat/web/.content/20_Aigua/02_infraestructures/05_estacions_depuradores_daigues_residuals/Fitxes_EDAR/dgvc_edar_gava_viladecans.pdf for Gavà WWTP.
All other relevant data, from both river water and WWTPs, are publically available at the Catalan Water Agency (ACA) website: http://aca.gencat.cat.

Location	Specific location, indicating latitude and longitude, of each sampling point of the two rivers and the two WWTPs is stated in the Supplementary Material provided with the manuscript. Likewise, the sampling point of all bacterial isolates obtained is specified in the Supplementary Material, and sampling location for E. coli isoaltes from this study and from other works included in the analysis of the current study can be graphically visualized at: https://microreact.org/project/a9-1ZIze0.<br>Furthermore, other relevant parameters related with sampling location can be consulted at the different interactive applications publically available at the Catalan Water Agency (ACA) website: http://aca.gencat.cat.<br>- For WWTPs, data are available at: http://sig.gencat.cat/visors/VISOR_ACA.html#param=param&color=vermell&background=topo_ICC&BBOX=144808.774834,4485000,649191.225166,4752000&layers=AIGUA_DEPURADORES<br>- For rivers, data are available at: http://aca-web.gencat.cat/WDMA/cercarDiagnostics.do
Access and import/export	The access to the sampling points, as well as the collection of the samples, was carried out under the pertinent regulations issued by the competent local authorities of the Catalan Government (Generalitat de Catalunya) and the responsible administrators of the Catalan Water Agency (ACA). The procedure was performed following all established requirements and considerations.
Disturbance	No disturbances were caused during the compilation of the samples, as the interventions were performed one single time for each sampling point, collecting a restricted volume of water/sediment samples and following a conscientious procedure.

Reporting for specific materials, systems and methods

We require information from authors about some types of materials, experimental systems and methods used in many studies. Here, indicate whether each material, system or method listed is relevant to your study. If you are not sure if a list item applies to your research, read the appropriate section before selecting a response.

Materials & experimental systems

n/a	Involved in the study
☒	☐ Antibodies
☒	☐ Eukaryotic cell lines
☒	☐ Palaeontology
☒	☐ Animals and other organisms
☒	☐ Human research participants
☒	☐ Clinical data

Methods

n/a	Involved in the study
☒	☐ ChIP-seq
☒	☐ Flow cytometry
☒	☐ MRI-based neuroimaging
